# Detection, Prevention, and Treatment of Asymptomatic Myxoma in a Young Adult Patient: A Case Report

**DOI:** 10.7759/cureus.81649

**Published:** 2025-04-03

**Authors:** Yuta Murai, Kouki Nakashima

**Affiliations:** 1 Department of Cardiovascular Surgery, Sagamihara Kyodo Hospital, Kanagawa, JPN

**Keywords:** cardiac murmur, cardiac myxoma, cryoablation, school health check-up, ventricular outflow tract

## Abstract

We present a case of myxoma originating from the right ventricular outflow tract. The patient was referred to our clinic after detecting a heart murmur at a routine school medical check-up, which is unique to Japan. He was treated with tumor resection and cryoablation to prevent recurrence. Cryoablation after tumor removal has the potential to prevent the recurrence of cardiac tumors. This case further emphasized the importance of regular school medical check-ups because it prevented sudden death.

## Introduction

Cardiac myxoma is a benign cardiac tumor, with an annual incidence rate of 0.5 to one case per one million people [1-4]. Depending on the location and the size of the tumor, patients with cardiac myxoma may present with different signs and symptoms [5], although cases of sudden death without symptoms have been reported [6]. Cardiac myxoma most commonly originates from the left atrium (72% to 92%), whereas right ventricular myxoma, similar to that in the present case, is the rarest, occurring in only 0.7% to 2.5% of cases [5].

Japan has a unique system of universal health insurance, where all citizens are covered by one type of medical insurance. In accordance with the School Safety and Health Law, all students must undergo a routine medical check-up every year. Cardiac examinations are conducted periodically in students to promote general health and prevent sudden deaths by early diagnosis of cardiac events, including obstruction and arrhythmias [7,8].

Here, we report the case of a 19-year-old male patient who was diagnosed with myxoma in the right ventricular outflow tract after being referred to our hospital due to the identification of a cardiac murmur during a school medical check-up. We performed tumor resection and cryoablation to prevent recurrence.

## Case presentation

**Figure 1:**
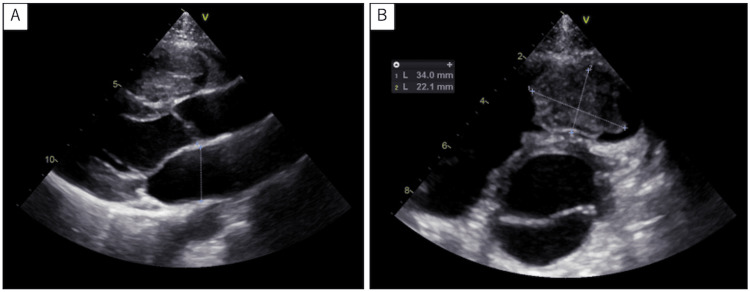
Echocardiographic view of the tumor in right ventricular outflow tract A. Parasternal long-axis tomogram and echocardiographic view of the tumor in the right ventricular outflow tract; B. Short-axis tomogram of aortic valve level and echocardiographic view of the tumor in the right ventricular outflow tract

**Figure 2:**
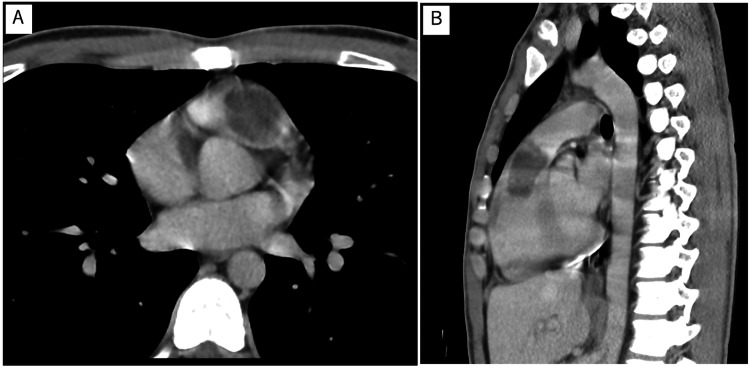
CT images of the tumour in the right ventricular outflow tract Axial (A) and sagittal (B) views of the tumor in the right ventricular outflow tract on CT. CT: computed tomography

**Figure 3:**
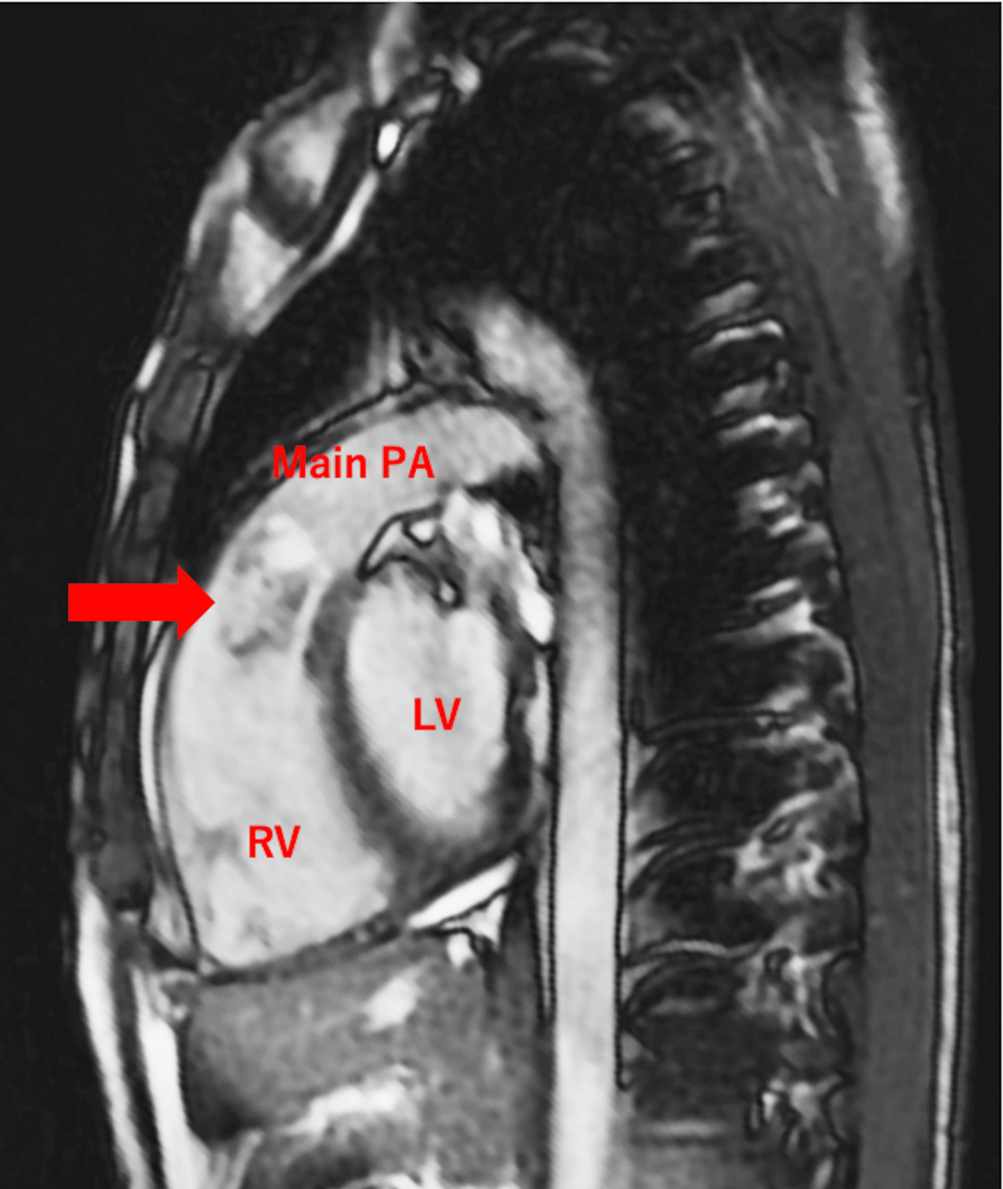
Sagittal view of the MRI showing the tumor in the right ventricular outflow tract The red arrow indicates the tumor in the right ventricular outflow tract, revealing high signal intensity within the tumor on T2-weighted imaging, reflecting the mucin contents of the tumor. MRI: magnetic resonance imaging; Main PA: main pulmonary artery; RV: right ventricle; LV: left ventricle

A 19-year-old male patient was referred to our hospital after a systolic ejection murmur was auscultated at the left sternal border in the second intercostal space during a school medical check-up. He did not complain of any specific symptoms. Medical history and family history were negative. Physical examination and blood tests were performed, including the evaluation of hormone and interleukin (IL)-6 levels, which were 2.4 pg/mL and within the normal range. Specifically, growth hormone (GH) and insulin-like growth factor 1 (IGF-1) levels were within normal ranges. No abnormalities were observed on the chest X-ray and electrocardiography. Echocardiography revealed normal left ventricular and valvular function. However, echocardiography and computed tomography (CT) showed a mass (44 mm × 21 mm) in the right ventricular outflow tract; echocardiography revealed tumor mobility (Figures 1a, 1b, 2a, 2b). No findings suggestive of distant metastasis were observed. Magnetic resonance imaging (MRI) showed high signal intensity within the tumor on T2-weighted imaging, reflecting the mucin of the tumor (Figure 3). Based on these findings, cardiac myxoma was diagnosed. Prompt surgery was planned because of the risk of sudden death due to obstruction of the right ventricular outflow tract in this patient.

**Figure 4:**
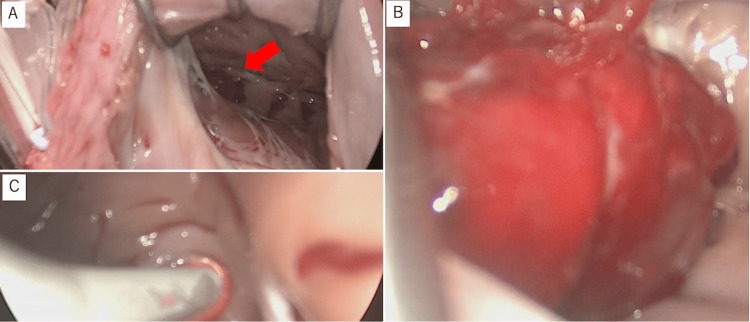
Surgical treatment of the myxoma A. Intraoperative view, the red arrow indicates the tumor from the right atrium into the right ventricle; B. Intraoperative view of the entire tumor after pressing on the right ventricular outflow tract from the outside of the heart; C. Cryoablation was performed at the root of the tumor.

The surgery was performed through median sternotomy under cardiopulmonary bypass with cardioplegic arrest. Through a right atrial incision, part of the tumor was visible in the right ventricular outflow tract over the opening of the tricuspid valve (Figure 4a). Pressing on the right ventricular outflow tract from the outside revealed the entire tumor. The tumor appeared to originate from the free wall of the right ventricular outflow tract (Figure 4b). The tumor was resected, and the broad pedicles, including the endothelial layer, were excised. Cryoablation was then performed to remove any potential remnant tissue using a flexible probe (CryoICE; AtriCure, Inc., Cincinnati, OH, USA) to extirpate remnant tissue. During this procedure, a target temperature of -70°C was maintained for 2 minutes at the base of the pedicle to ensure that the entire resected area underwent cryoablation (Figure 4c). The cardiopulmonary bypass time was 117 minutes; the cardiac arrest time was 71 minutes.

**Figure 5:**
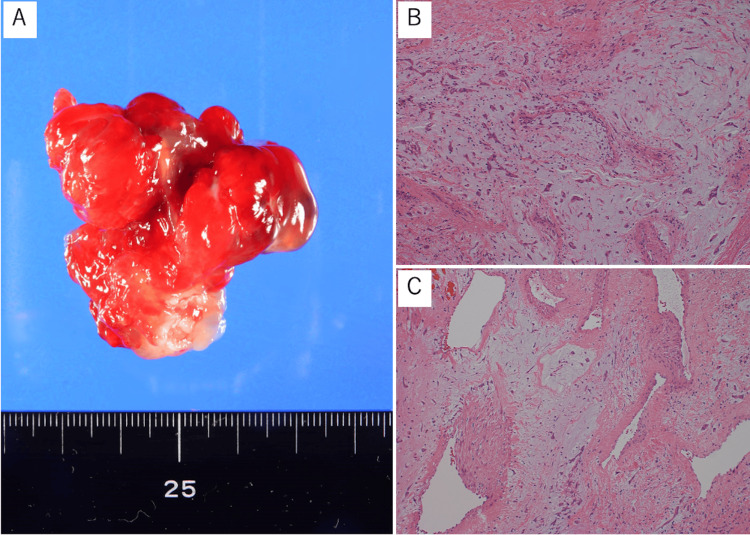
Macroscopic and histopathological findings of cardiac myxoma A. Resected myxoma; the maximum diameter is 4 cm; B. Spindle-shaped tumor cells are interspersed within a myxoid stroma. There are no malignant findings. (hematoxylin and eosin staining; bar = 100 μm; magnification × 200); C. Bleeding and thrombus formation are seen in the tumor (hematoxylin and eosin staining; bar = 100 μm; magnification × 200).

**Figure 6:**
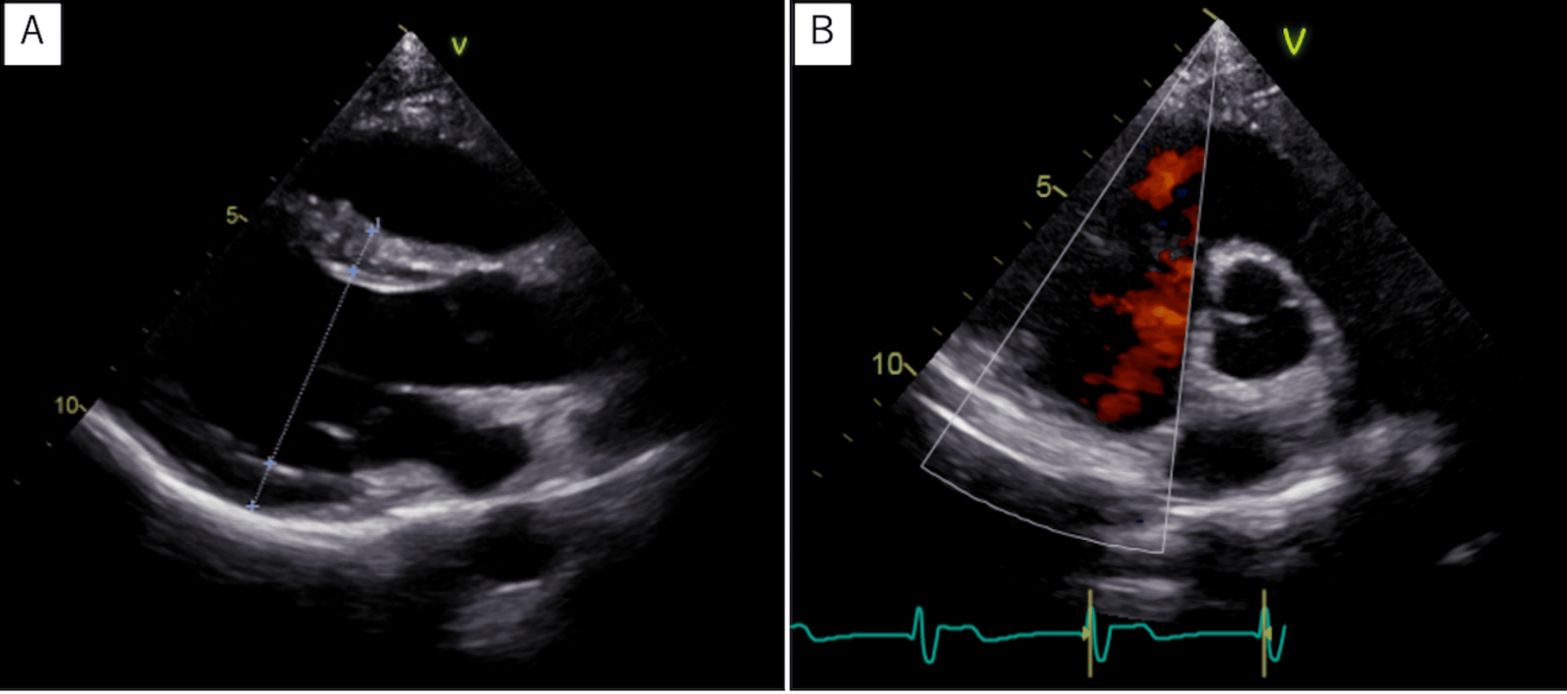
Postoperative echocardiographic view of the tumour in the right ventricular outflow tract A parasternal long axis tomogram (A) and a short-axis tomogram of the aortic valve level (B) show no residual tumor.

Pathological examination of the removed tissue reported a typical myxoma tissue (Figures 5a-5c). The postoperative course was favorable. The follow-up echocardiography after the operation was as good as the preoperative findings, with no decrease in cardiac function or appearance of valve regurgitation, and no arrhythmias were detected. The patient was discharged home without any complaints. Five years after surgery, no residual tumor was detected. (Figures 6a, 6b). Postoperatively, no findings suggestive of metastasis were observed either.

## Discussion

We performed prompt tumor resection and cryoablation to prevent right ventricular outflow tract myxoma recurrence in a patient with a heart murmur detected during a school medical check-up.

The School Safety and Health Law of Japan’s unique universal health insurance system stipulates that all students should undergo annual school medical check-ups, including cardiac examinations. In 1995, electrocardiography was made compulsory for all elementary and high school students upon school enrollment. This unique large-scale school cardiac screening system has been instrumental in markedly reducing the incidence of sudden cardiac death among elementary and secondary school students in Japan, from 0.60 per 100,000 in 1987 to 0.08 in 2018 [7,8]. In asymptomatic cases, such as ours, a tumor may grow unnoticed, with a risk of sudden death [6,9]. Detection of a heart murmur during a routine school medical check-up in this student prevented such an outcome. 

In a previous study, cardiac myxoma was reported to recur in 5.6% of patients, with younger age at surgery, smaller tumor dimension, and tumor localized to the ventricles considered as predictors of recurrence [10]. Inadequate resection may also lead to recurrence. In this case, the young age of 19 years and the fact that the tumor was confined to the ventricles indicated a high risk of recurrence. There have been several previous reports on cryoablation for the prevention of recurrence in cardiac myxomas with good outcomes [11-15]. There are several methods for preventing cardiac myxoma recurrence. Wide free margin excisions may lead to complications due to anatomical defects resulting from extensive resection. Furthermore, performing resections near valves or fragile tissues can also be difficult [16]. Another approach for recurrence prevention involves coagulating the resection lines with electrocautery [17]. However, the extent of required electrocautery remains unclear; there is also a risk of tissue loss. At our institution, we perform cryoablation on the pedicle after myxoma resection, as it allows for destroying residual tumor cells without excessive tissue removal while preserving the tissue morphology. Additionally, in this case, a five-year postoperative follow-up was conducted, revealing no recurrence. Wherever there is a possibility of recurrence, measures should be taken to prevent recurrence, and cryoablation of the remaining tissue should be considered, as in this case.

Interleukin-6 is involved in tumor growth and angiogenesis in patients with cardiac myxomas; a correlation between tumor size and IL-6 levels has been observed. Additionally, IL-6 has been suggested as a potential marker for cardiac myxoma recurrence [18,19]. In the present case, no elevation in IL-6 levels was observed preoperatively. Moreover, there was no increase in IL-6 levels even five years post surgery. To enable early detection of recurrence, our postoperative follow-up protocol for cardiac myxoma includes cardiac ultrasound, chest X-ray, electrocardiography, and blood tests, including those for IL-6, every three months within the first year after surgery to screen for any signs of recurrence. Thereafter, we perform annual follow-up examinations, including cardiac ultrasound, blood tests, electrocardiography, and chest X-ray.

## Conclusions

Early detection of cardiac myxoma is critical to preventing life-threatening complications such as sudden cardiac death. In this case, a routine school medical check-up led to the identification of a heart murmur, ultimately allowing for timely diagnosis and surgical intervention. Given the potential risk of recurrence, cryoablation was performed in addition to tumor resection, ensuring complete removal of remnant tissue. This case highlights the importance of regular school medical check-ups in detecting asymptomatic cardiac conditions and emphasizes the role of cryoablation as a preventive measure against myxoma recurrence.
